# A disaster of politics: The energy supply crisis in South Africa

**DOI:** 10.4102/jamba.v15i1.1492

**Published:** 2023-10-18

**Authors:** Gideon J. Wentink

**Affiliations:** 1 Unit for Environmental Sciences and Management, Faculty of Natural and Agricultural Sciences, North-West University, Potchefstroom, South Africa

**Keywords:** disaster, South Africa, state of disaster, declaration of disaster, energy supply crisis, politics

On 09 February 2023, the South African government declared and classified the energy supply crisis in South Africa as a state of disaster. This is directly the opposite of what the president announced a few months before the event (Ramaphosa 2022). To answer the question posed in the title of this opinion piece, it is necessary to take a brief look at the build-up to the declaration and classification of the state of disaster of 09 February 2023. Then I will discuss what a disaster entails according to South African legislation, and I will conclude with closing comments.

## How did we come to where we are?

The energy crisis in South Africa did not arise overnight. Indeed, it has been a quarter of a century in the making. The South African government was warned in 1998 by the Department of Minerals and Energy that by 2007, the generation surplus of the country’s electricity public utility, known as Eskom, would be fully utilised (DME 1998:41). It cautioned that steps would have to be taken to ensure that demand did not exceed available supply capacity and that ‘appropriate strategies’, including those with long lead times, were implemented in time (DME 1998:53).

The consequences have been devastating. Since 2007, Eskom – which has a *de jure* monopoly on the generation of power in South Africa (Van Niekerk 2020:3–4) – has frequently been unable to generate sufficient power to meet demand. In order to avoid a total collapse of the electricity grid, Eskom introduced rolling blackouts, better known as ‘loadshedding’.

The most recent period of sustained loadshedding began in March 2021 and has persisted relatively continuously to date (February 2023), with particularly long periods of stage 6 loadshedding (where electricity supply would be off for 11.5 h per day) dominating the months of June and July 2022. The crisis was further exacerbated by illegal or unprotected strike action in June 2022 (Eskom 2022). Such strike action is one that does not comply with the requirements stipulated in the *South African Labour Relations Act* (Erasmus 2021) and has in the past been a frequent cause of loadshedding and a failure to perform vital maintenance and other mandatory upkeep operations. In response to the escalating crisis, President Cyril Ramaphosa announced that ‘we do not need a state of emergency or a national disaster to implement common sense regulations that should help in resolving our energy crisis’ (Ramaphosa 2022).

**FIGURE 1:**
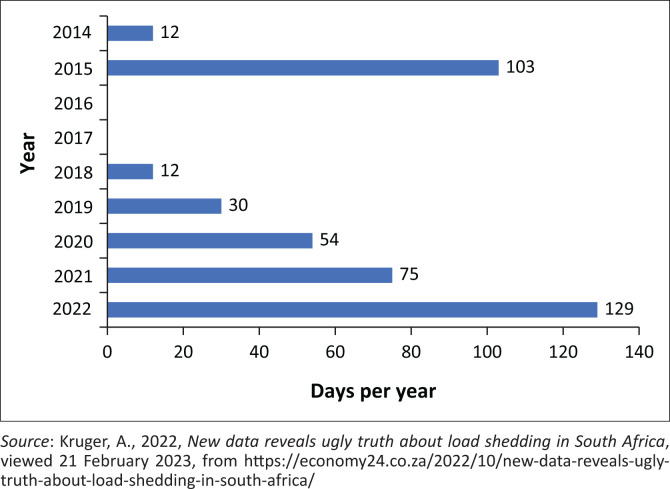
Number of days of loadshedding in South Africa per year.

According to Kruger (2022), the incidence of loadshedding and unplanned maintenance has increased steadily, while the ‘Energy Availability Factor’ has decreased steadily. This reflects an increase in breakdowns at power plants. As can be seen in Figure 1, the number of days of loadshedding per year has steadily increased from year to year. Up to 31 July 2023, South Africans have had a single day without loadshedding (for 2023), and the prediction in January was that electricity will be rationed for 250 days in 2023, which will be more than any previous year (Naidoo 2023). While the outlook is dire, that is not determinative of whether the circumstances warrant the classification and declaration decisions.

The current energy crisis is not the result of environmental or other extraneous factors beyond the control of Eskom and the state; it is entirely self-inflicted. Corruption, looting, and fraudulent dealings are part and parcel of Eskom’s collapse. Even though Olson (2000:272–273) argues that disasters are political occasions because their implications must be managed and explained, the energy supply crisis in South Africa is the result of corruption and ineptitude as will be made clear in the following paragraphs.

Policy indecision, political agendas, and a lack of forward-planning have had an equally deleterious effect on Eskom’s operations. This includes the hollowing out of Eskom’s skill pool and the removal of experts, established managers and labourers with institutional knowledge (Marrian 2023). The South African Parliament’s Standing Committee on Public Accounts (SCOPA), which oversees the financial statements of all government departments and state institutions, also gives concrete examples of the type of issues that have plagued Eskom for the past two decades (SCOPA 2022).

Paragraph 1.3 of SCOPA’s (2022) report notes the scale of fruitless and wasteful expenditure at Eskom:

Before the actual visit, the Committee received an audit briefing from ESKOM’s auditing firm SNG for the financial year 2020/2021. Medupi incurred irregular expenditure of R355 million for the financial year 2020/2021, this being due to irregular tender processes and breach of legislation, which included modifications exceeding National Treasury thresholds. For Kusile, irregular expenditure amounting to R27 million and was attributed to the same reasons as for Medupi. Fruitless and wasteful expenditure of R4.4 billion was incurred by Medupi and Kusile. R1 billion of which was an overpayment to ABB by Kusile. ABB entered into a settlement agreement with ESKOM to pay about R1 billion. Further overpayments estimated at R2.5 billion were disclosed as matters under investigation, following supplier claims which could not be substantiated. (p. 2)

Paragraph 4.1.1, on the other hand, highlights the cost of incompetence at Eskom with reference to the well-publicised incident last year at Medupi (SCOPA 2022):

Unit 4 suffered an explosion in November 2021, resulting in damages estimated at R2.4 billion, crippling the total output of electricity by about 700 MW It was reported that there was no visible physical damage on the turbine rotors and other components, but these were nonetheless taken away for testing and refurbishment. The explosion occurred largely due to procedure not having been followed, and due to lack of proper supervision, when air was let into the generator prematurely while there was still hydrogen in the generator. This Unit 4 was said to be expected to be fully operational again by August 2024. An investigation into the matter is being concluded, and 9 employees – 2 senior managers, 3 shift managers and 4 operators – were charged. (p. 3)

Relating to the fruitless and wasteful expenditure, the Zondo Commission of Inquiry into State Capture provides insight with the following quote in respect of looting and corruption at Eskom (Zondo 2022):

In total, R14.7 billion of Eskom’s contracts are calculated to have been afflicted by State Capture according to the Flow of Funds’ investigation and, of this, McKinsey’s MSA and Corporate Plan contracts account for R1.1 billion, and related payments to Trillian account for R595.2 million from these two contracts. (p. 990)

Further issues include Eskom’s failure to tackle so-called ‘evergreen’ tender contracts, slow progress with constructing new capacity despite large amounts of money being spent, incompetence and a failure to implement SCOPA recommendations from previous visits (SCOPA 2022).

From the aforementioned, it becomes evident that blame for the energy crisis must be placed at the door of the state as the sole shareholder in Eskom (2023). Significantly, the crisis has not arisen gradually or progressively, but rather sporadically over the past two decades directly because of either poor policy, perennial fraud and corruption, factionalism and in-fighting among the executive and management, and poor planning generally (Crompton 2019).

## What does legislation and the framework say?

The definition of disaster, according to the *Disaster Management Act* (2002/57), requires that there be an ‘occurrence’. This is echoed in section 23(1) of the Act, which provides that:

When a disastrous event occurs or threatens to occur, the National Centre must, for the purpose of the proper application of this Act, determine whether the event should be regarded as a disaster in terms of this Act. (*Disaster Management Act* 57 of 2000, s. 23[1])

The classification and declaration decisions were not taken in response to any such occurrence or threatened occurrence. Rather, both decisions were taken in response to ‘the substantial impact caused by the severe electricity supply constraint’ (South Africa 2023). Indeed, it is strictly the ‘impact of the severe electricity supply constraint’ which the Head of the National Centre has classified as a national disaster. But that impact does not, in and of itself, constitute an occurrence or a threatened occurrence. It is, at best, the consequence of an occurrence. It therefore does not, and cannot, constitute a disaster for purposes of the Act.

In any event, even if there was an occurrence or a threat of an occurrence as required by the Act, it plainly would not constitute a disaster in the sense contemplated by the Act. The definition of ‘disaster’ does not cater for self-inflicted systemic failure resulting from mismanagement, corruption and policy uncertainty.

The declaration is not in reaction to a ‘disaster’ in order to mitigate its effects and assist those who are affected beyond their means to effectively respond. Rather, it is an attempt to remedy an infrastructure and service delivery crisis, which has been decades in the making.

The prevailing power crisis cannot be described as an ‘occurrence’ or ‘event’. It is better described as a situation that can with time either improve or deteriorate. It would be impossible to objectively ascertain at what point in time the energy crisis became, or will become, a ‘disaster’. Rather, the energy crisis is a confluence of separate yet interrelated factors and events, which factors came into existence or became problematic at different times, with differing levels of severity, and were influenced by certain events or occurrences.

Most importantly, no single ‘event’ or occurrence within the National Disaster Management Framework (NDMF) (South Africa 2005) would necessarily constitute a ‘disaster’ in and of itself. It is only when viewed holistically and defined together that the various factors constitute the actual energy ‘crisis’ – which, per definition, cannot be an ‘occurrence’.

A further qualification is the fact that a crisis may evolve and change, whereas a disaster occurs once and finally, after which the consequences or ramifications may prove to be multifaceted or fluid or engender crises of their own. A simple example would be that while the lack of generating capacity is a crisis, the collapse of the entire grid would be a national disaster, as it would be an occurrence – despite being anticipated, manmade, and progressive in onset.

Where the NDMF refers to infrastructure, it is mentioned almost exclusively in the context of damage to infrastructure. For instance, there is reference to an ‘infrastructure collapse’ as a type of disaster that comes closest to describing the present situation, but importantly, the risk of the grid collapsing is entirely remote.

The declaration of the national state of disaster has not been accompanied by any meaningful details of the measures to be put in place (South Africa 2023). It therefore does not appear to serve a bona fide purpose. Rather, the declaration appears to have resulted from political pressure and improper influence. In January 2023, less than a month before the classification and declaration decisions, the executive body of the ruling party, the African National Congress, had decided that a state of disaster was necessary (ANC 2023).

## Concluding remarks

In light of the aforesaid discussion, it becomes evident that the energy supply crisis in South Africa is not a disaster but is in fact the result of corruption and incompetence in the South African government as well as the electricity supplier for the country. I am of the opinion that there are politics at play, which fall outside of the scope of this manuscript, but which have significantly influenced the decision to wrongfully declare the state of disaster. This does not do the democratic process any good, especially in South Africa, which prides itself on its democratic record since 1994.
